# Assessment of *E. coli* Expression System for Overexpression of Active Recombinant Ocriplasmin

**DOI:** 10.34172/apb.2021.065

**Published:** 2020-06-21

**Authors:** Roghayyeh Baghban, Safar Farajnia, Younes Ghasemi, Mojtaba Mortazavi, Naser Samadi, Nosratollah Zarghami

**Affiliations:** ^1^Medical Biotechnology Department, Faculty of Advanced Medical Science, Tabriz University of Medical Sciences,Tabriz, Iran.; ^2^Research Committee, Tabriz University of Medical Sciences, Tabriz, Iran.; ^3^Biotechnology Research Center, Tabriz University of Medical Sciences, Tabriz, Iran.; ^4^Drug Applied Research Center, Tabriz University of Medical Sciences, Tabriz, Iran.; ^5^Department of Pharmaceutical Biotechnology, Faculty of Pharmacy and Pharmaceutical Sciences Research Center, Shiraz University of Medical Science, Shiraz, Iran.; ^6^Department of Biotechnology, Institute of Science and High Technology and Environmental Science, Graduate University of Advanced Technology, Kerman, Iran.

**Keywords:** Ocriplasmin, Recombinant expression, Vitreomacular adhesion (VMA)

## Abstract

**Purpose:** Ocriplasmin (Jetrea TM) is a FDA approved recombinant enzyme utilized in the treatment of vitreomacular adhesion (VMA). This is a recombinant C-terminal fragment of human plasmin produced using yeast Pichia pastoris. Since ocriplasmin does not contain any Oor N-glycosylation or some other post-translational modifications, bacterial expression systems such as Escherichia coli could be considered as an economical host for recombinant expression. In the present study, we aimed to evaluate the efficiency of *E. coli* expression system for highlevel expression of recombinant ocriplasmin.

***Methods:*** The gene coding for ocriplasmin was cloned and expressed in *E. coli* BL21. The bacterial cells were cultured on large scale and the expressed recombinant protein was purified using Ni-NTA chromatography. Refolding of denatured ocriplasmin to active enzyme was carried out by the stepwise removal of denaturant. The identity of recombinant ocriplasmin was confirmed using western blotting and ELISA assays. The presence of the active ocriplasmin was monitored by the hydrolytic activity assay against the chromogenic substrate S-2403.

***Results:*** The final yield of *E. coli* BL21-produced ocriplasmin was approximately 1 mg/mL which was greater than that of *P. pastoris*. Using western blotting and ELISA assay, the identity of recombinant ocriplasmin was confirmed. The hydrolysis of chromogenic substrate S-2403 verified the functional activity of *E. coli* produced ocriplasmin.

***Conclusion:*** The results of this study indicated that *E. coli* could be used for high level expression of ocriplasmin. Although the recombinant protein was expressed as inclusion body, the stepwise refolding leads to the biologically active proteins.

## Introduction

Ocriplasmin, also known as microplasmin, represents a novel pharmacologic agent approved by the Food and Drug Administration. It is a recombinant truncated human plasmin which is used for the treatment of vitreoretinopathies including symptomatic VMA.^1-3^ Abnormal symptomatic VMA is an age-related disorder that occurred due to partial PVD. This can exert traction on the retina and may result in visual distortions and even macular holes that in more serious cases leads to loss of vision.^4^ Till the FDA approval of ocriplasmin, the only treatment for VMA was a surgical method as vitrectomy. This method although has the risk of retinal damage,^5,6^ that describes why some of proteolytic enzymes, containing plasmin, have been experienced for enzymatic release of retinal traction.^7-10^ Ocriplasmin is a nonsurgical treatment option to induce posterior vitreous detachment due to its potential for degradation of fibronectin and laminin after intravitreal injection.^11-14^

Ocriplasmin, contains two polypeptide chains of 230 and 19 residues which attached by two disulfide bonds. Early, it was found that ocriplasmin has restricted stability after injection into the vitreous, while stable in its formulation buffer.^15^ The assessment of therapeutic efficacy provided after the expression and purification of recombinant ocriplasmin in the *Pichia pastoris*. Ocriplasmin is expressed as a precursor followed by activation using recombinant staphylokinase or urokinase.^16^ Since the ocriplasmin does not contain O- or N-glycosylation or some other post-translational modifications, bacterial expression systems such as *E. coli* is a valuable host for the production of this therapeutic protein. On the other hand, compared to *E. coli*, production of recombinant protein in *P. pastoris* is more expensive and time-consuming. Numerous FDA approved therapeutic recombinant proteins are produced in *E. coli* expression system. Abundant advances have recently been happened for adjusting these expression systems for easy and high level production of therapeutic proteins.^17^ In the present study, we aimed to assess the efficiency of *E. coli*expression systemfor high level recombinant ocriplasmin expression.

## Materials and Methods

### Gene amplification and cloning

The gene encoding of human ocriplasmin was amplified using the gene-specific primers. The polymerase chain reaction (PCR) protocol (30 cycles) was performed with an initial denaturation for 4 min at 94°C, annealing for 45 seconds at 55^o^C, extension for 45 seconds at 72°C and final extension for 5 min at 72°C. The PCR product was evaluated on 1% agarose gel and purified using FavorPrep^TM^ GEL/PCR purification mini kit according to the manufacturer’s instructions. After purification, PCR product and vector (pET-28a) were digested by *SlaI* and *NdeI*. Compatible cohesive ends of the digested vector and PCR product were ligated at 16^o^C overnight. The ocriplasmin gene was cloned in the pET-28a vector.

***E. coli*** **transformation and selection**

The expression construct (pET-28a-ocriplasmin), containing the bacteriophage *T7* promoter, was transformed into the *E. coli DH5α strain*by heat shock transformation procedure.For the selection of desired recombinant clones, the transformants were plated onto LB/kanamycin (100 mg/mL) medium. After 16-hour incubation at 37°C some colonies appeared on LB-plate. Transformants were screened and the recombinant right clones were further confirmed by PCR.

### Expression of recombinant ocriplasmin in E. coli

A single clone containing PET-28a-ocriplasmin construct was selected for the expression of recombinant ocriplasmin in LB/kanamycin (100 mg/mL) medium. The plasmid was extracted and then transformed into *E. coli BL21 (DE3)* as expression strain. Expression of positive clone was accomplished at OD600 of 0·5 with 1mM IPTG at 37°C for 24 hours. The bacterial cells were centrifuged for 10 minutes at 9000 rpm, and after that, the production of recombinant ocriplasmin was evaluated on 12% SDS-PAGE gel.

### Isolation and solubilization of inclusion bodies

The pellet was resuspended in the sonication buffer (100mM NaH2Po4, 10 mM Tris, pH 10) and sonicated for 30 seconds and 30 times on the ice. Then, the sample was centrifuged at 9000 rpm at 4ºC for 10 minutes. To solubilize isolated inclusion bodies, we used 100mM NaH_2_Po_4_, 10mM Tris, 8M urea and 10mM imidazole, pH 8. The solution was then shaken for 60 minutes at room temperature. After centrifugation at 10000 rpm for 10 minutes, the amount of protein in the supernatant (soluble fraction) was estimated by nanodrop assay and 12% SDS-PAGE gel.

### Purification of recombinant ocriplasmin

After solubilization of inclusion bodies, protein extract was loaded on Nickel-chelate affinity chromatography according to the approach offered by the company producers. The recombinant protein was eluted with elution buffer (NaH_2_Po_4_ 50mM, NaCl 300mM, imidazole 250mM, pH 8), after washing by washing buffer (NaH_2_Po 50mM, imidazole 10mM, NaCl 300mM, pH 8). The eluted recombinant proteins were accumulated in 10 microtubes and the results were analyzed by 12% SDS-PAGE gel.

### Refolding of denatured ocriplasmin

Refolding of the denatured recombinant protein was performed using serial dialysis at 4^°^C in the following buffers for 6 days. first day: NaH_2_Po_4_ 100mM, Tris 10mM, dithiothreitol (DTT) 5mM and urea 6M, pH= 8; second day: NaH_2_Po_4_ 100mM, Tris 10mM, DTT 2mM, urea 4M, arginine 400 mM, GSH (reduced Glutathione ) 5mM, and GSSG (oxidized glutathione ) 1mM, pH= 8; third day: NaH_2_Po_4_ 100mM, Tris 10mM, DTT 1mM, urea 2M, arginine 400 mM, GSH (reduced Glutathione) 5mM, and GSSG (oxidized glutathione) 1mM, pH= 8; fourth day: NaH_2_Po_4_ 100mM, Tris 10mM, urea 1M, arginine 400 mM, reduced GSH 2.5mM, and GSSG 0.5mM, without DTT pH= 8; fifth day: NaH_2_Po_4_ 100mM, Tris 10mM, arginine 400 mM, reduced GSH 2.5mM, and GSSG 0.5mM, without urea and DTT, pH= 8; sixth day: NaH_2_Po_4_ 100mM, Tris 10mM, arginine 200 mM, without urea, DTT, reduced GSH and GSSG.^18^

### SDS-PAGE and Western blot analysis

The purified ocriplasmin, under reducing condition, was evaluated by 12% SDS-PAGE gel and Coomassie Blue staining. For western blotting, the recombinant proteins were transferred to the PVDF (polyvinylidene fluoride) membrane for 45 minutes at 30 mA. Then, the PVDF membrane was blocked with 5% skim milk in PBS at 4°C as overnight. The PVDF membrane was incubated with 1: 3000 dilutions of an anti-ocriplasmin antibody for 1 hour. The PVDF membrane was then washed and exposed to a HRP (horseradish peroxidase)-conjugated secondary antibody (1/5000 dilution) for 1 hour. Finally, the membrane was exposed to ECL for high-sensitivity western blot detection and visualized by an imaging system.

### Dot blotting analysis

Ten micrograms of purified ocriplasmin produced in *Bl-21* was transferred to the PVDF membrane. Then, the membrane was dried and blocked by 5% BSA or skim milk diluted in PBST. After 16 hours the membrane containing ocriplasmin was washed and incubated with 10 µg/mL of anti-ocriplasmin antibody solution for 60 minutes at room temperature with gentle shaking. Antibody–antigen complexes were detected with an anti-rabbit antibody (1/5000 dilution) solution and ECL as the substrate.

### Specificity determination

The specificity of recombinant ocriplasmin produced in *E. coli* was determined by enzyme-linked immunosorbent assay (ELISA). The microplate was coated with 10 μg of purified ocriplasmin and incubated overnight, at 4°C. Using PBST (PBS+ 0.05% Tween 20), the wells were washed and then blocked with 3% BSA in PBS for 1 hour at room temperature followed by washing with PBST. One hundred microliters from different dilutions (1/400, 1/800, 1/1600, 1/3200, 1/6400, 1/12800, 1/25600) of serum containing antibody were added into the wells and incubated for 60 minutes at room temperature. After washing with PBST, the secondary antibody (anti-rabbit antibody conjugated to HRP (1/5000 dilution)) was added to the wells and incubated at room temperature for 1 hour. Finally, after washing the wells, 100 μL of TMB substrate was added and incubated at room temperature for 10–15 minutes. The enzyme reaction was stopped using 100 μL of 1N Sulfuric acid, and optical density (OD) was determined at 450 nm.

### Activation of zymogen form of ocriplasmin and hydrolytic activity assay

The purified precursor of ocriplasmin was converted to its catalytically active form using the urokinase enzyme (Sigma). 5–20 mM of zymogen form of ocriplasmin was incubated at 37°C in the presence of urokinase (the zymogen form of ocriplasmin/ urokinase ratio: 100/1). The appearance of the active recombinant ocriplasmin was monitored by the hydrolytic activity assay against the chromogenic substrate S-2403 as formerly illustrated.^15^ When the maximum activity was achieved, the extent conversion of ocriplasmin was evaluated using 12% SDS-polyacrylamide gel.

## Results and Discussion

**Figure 1:**
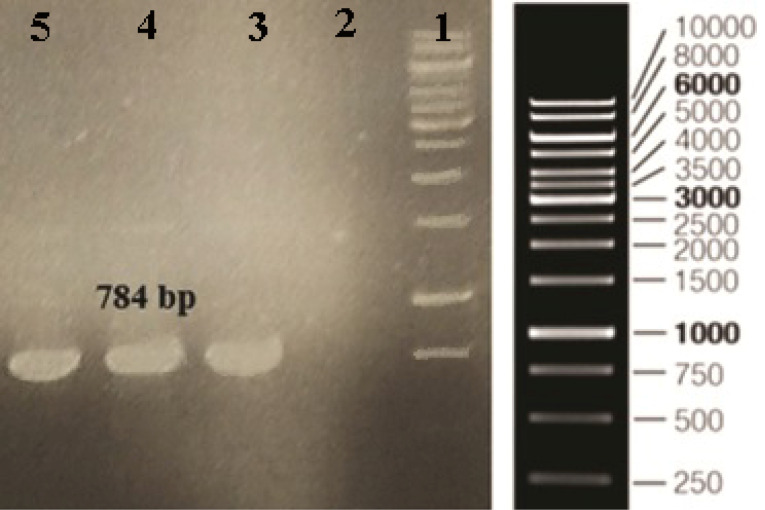
Confirmation of recombinant clones screened by PCR. Column 1: DNA ladder, Column 2: Negative control, Column 3-5: PCR products (784 bp).

Ocriplasmin (Jetrea) is the first nonsurgical alternative to vitrectomy for treating VMA and VMT. This enzyme degrades some structural proteins,^19^ such as fibronectin and laminin, which have an important role in the vitreoretinal attachment.^20,21^ Ocriplasmin includes just the enzymatic section of plasmin, whereas the other parts are deleted. Due to smaller size of ocriplasmin compared to the original molecule (27 kDa versus 80 kDa,) when retaining the similar enzymatic activity, theoretically may provide better permeation into epiretinal tissues.^22^ Commercially, ocriplasmin is produced using yeast *P. pastoris*. Since ocriplasmin does not contain any O- or N-glycosylation or some other post-translational modifications, *E. coli* can be considered as an economical and simple host for recombinant expression. Also, compared to* E. coli*, the production of recombinant proteins in *P. pastoris*is more laborious and time-consuming. *E. coli*, as the well-established cell factory, is the preferred organism and a perfect heterologous system of choice for the expression of recombinant proteins. In the present study, we evaluated *E. coli* expression system for the production of active ocriplasmin. The DNA sequence encoding the ocriplasmin gene was amplified by PCR and subcloned into the pET-28a vector. The vector containing the ocriplasmin gene was transformed into *DH5α* competent *E. coli*. After 16 hours’ incubation at 37°C, some colonies appeared on the LB kanamycin plate. Using colony PCR, the recombinant positive clones were confirmed (Figure 1).

**Figure 2:**
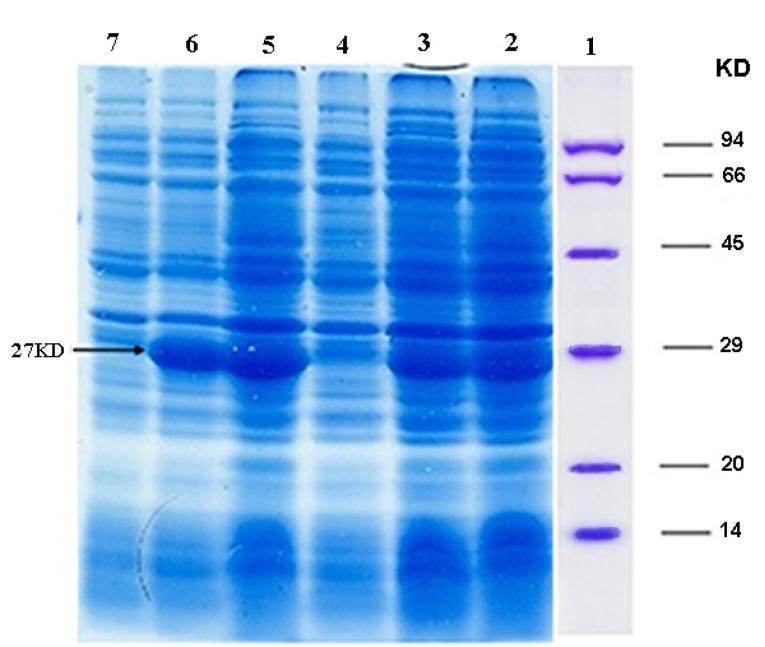
Expression of ocriplasmin in *E. coli BL21 (DE3)*strain, The samples were collected and analyzed by SDS-PAGE: Column 1: Protein marker, Column 2: a 27 kDa protein product induced by IPTG after 24 hours (clone 1), Column 3: a 27 kDa protein product induced by IPTG after 3 hours (clone 1), Column 4: Negative control (clone 1), Column 5: a 27 kDa protein product induced by IPTG after 24 hours (clone 2), Column 6: a 27 kDa protein product induced by IPTG after 3 hours (clone 2), Column 7: Negative control (clone 2).

**Figure 3:**
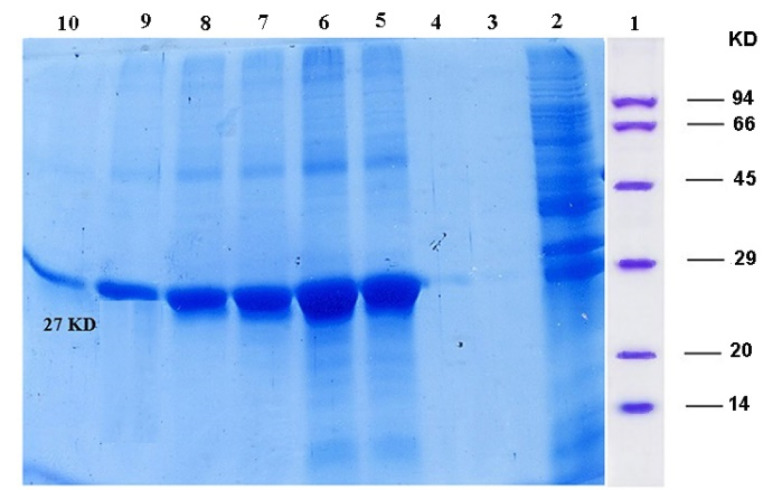
Recombinant protein purified using Ni-NTA column and analyzed by SDS-PAGE; Column 1: protein marker, Column 2: flowery, Column 3, 4: washing, Columns 5-10: purified ocriplasmin.

**Figure 4:**
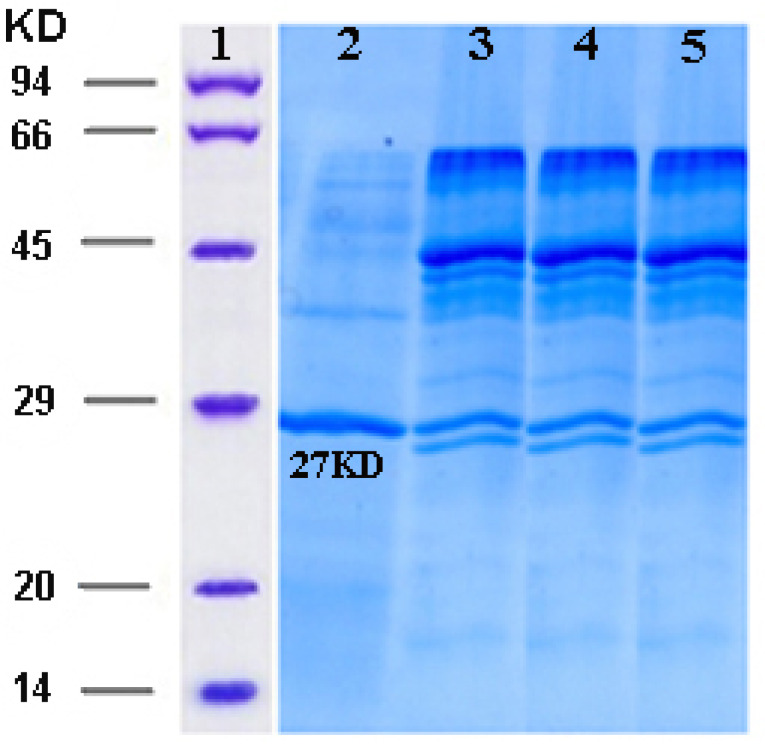
Activation of ocriplasmin by urokinase. Reducing SDS–PAGE illustrating that activation with urokinase is almost complete within 30 min. Ocriplasmin (27 kDa) is converted into two fragments upon activation. Column 1: Protein marker, Column 2: Ocriplasmin before activation, Column 3-5: Samples collected 10 min, 30 min, 60 min, after addition of 2% urokinase.

The expression of recombinant ocriplasmin was analyzed on SDS-PAGE (Figure 2). Figure 3 illustrates the purification of recombinant ocriplasmin using the Ni-NTA column. The results indicated that the protein yield from *E. coli* was 1 mg/mL in culture. This is greater than the yield of recombinant ocriplasmin in *P. pastoris*(0.5 mg/mL).^23^ Our results showed high-level expression of ocriplasmin as inclusion bodies in *E. coli*expression system.To achieve the biologically active ocriplasmin from the inclusion bodies, refolding of denatured protein was carried out by using serial dialysis at 4^°^C for 6 days in a buffer containing oxidized /reduced glutathione and L-arginine. The greater efficiency of this process can be attributed to the refolding enhancement efficiency of GSSG and L-arginine that has been reported in some previous studies.^24-28^ The zymogen form of ocriplasmin was converted into its active form using urokinase (Figure 4). The rate of zymogen conversion into the active form of ocriplasmin was followed via monitoring of hydrolytic activity of aliquots collected at different time intervals. The presence of the active ocriplasmin was monitored by the hydrolytic activity assay against the chromogenic substrate S-2403. It was indicated that under the experimental conditions used in this study, the activity attained a maximum after 30–60 minutes. Our results were similar to studies conducted by Noppen and Aert.^4,15^

**Figure 5:**
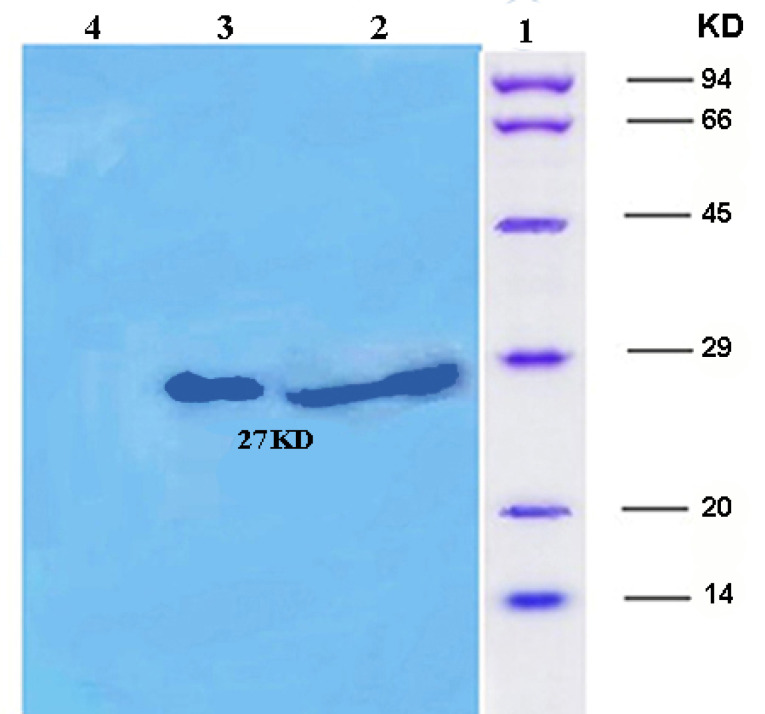
Western blotting: Column1: Protein marker, Column 2: Purified ocriplasmin expressed in *E. coli,*Column 3: a 27 kDa protein product induced by IPTG after 24 hours, Column 4: Negative control.

**Figure 6:**
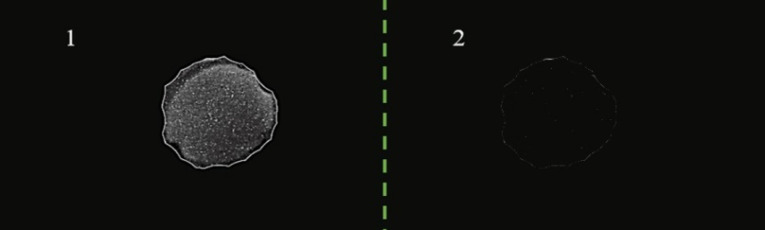
Dot blot analysis with HRP conjugated anti-rabbit antibody for confirmation of protein of interest. Column 1: Purified ocriplasmin expressed in *E. coli,* Column 2: Negative control.

**Figure 7:**
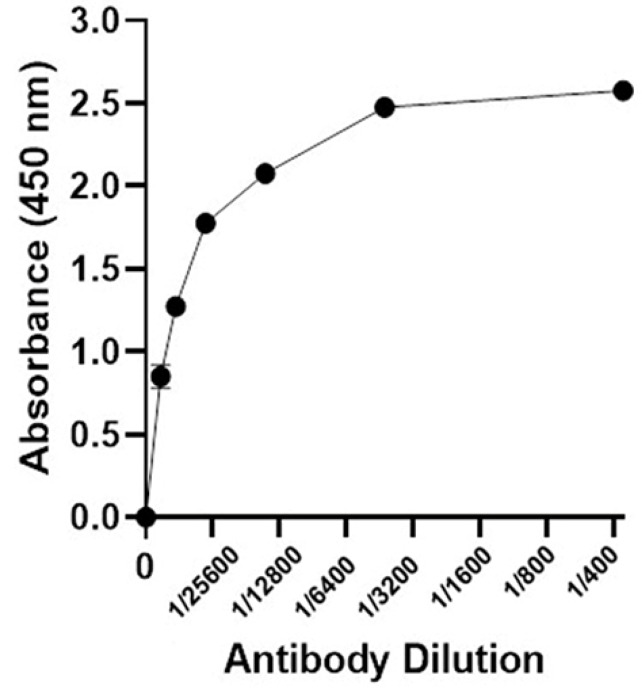
Elisa assay using anti-ocriplasmin antibody and recombinant protein.

Western blotting (Figure 5) and dot blot (Figure 6) was accomplished for the evaluation of the recombinant protein. The specificity of the ocriplasmin toward antibody was determined by ELISA assay at 450 nm (Figure 7). The ELISA was carried out with anti-ocriplasmin antibody raised in rabbit confirmed the functionality of the bacterial-expressed protein. The affinity of our protein was reasonable.

## Conclusion

The results of present study showed that ocriplasmin can be abundantly generated by *E. coli*. Analysis for activity by the chromogenic substrate S-2403 confirmed the hydrolytic activity of purified ocriplasmin which suggests the possibility of the use of bacterially expressed ocriplasmin in controlling the vitreoretinopathies and treating thrombotic diseases. The successful production of ocriplasmin in *E. coli -Bl21*lays a solid foundation for its possible future applications.

## Ethical Issues

This article does not contain any studies with human participants or animals performed by any of the authors.

## Conflict of Interest

The authors declare that they have no conflict of interest.

## Acknowledgments

The authors would like to acknowledge the financial support of the Biotechnology Research Center, Tabriz University of Medical Sciences, Tabriz, Iran, as Ph.D. thesis of R. Baghban. This work was also supported by the biotechnology development council.
